# The role of Met, Src and Stat3 in basal-like breast cancer invasion

**DOI:** 10.1186/1753-6561-7-S2-P21

**Published:** 2013-04-04

**Authors:** E Carefoot, L Raptis, PA Greer, BE Elliott

**Affiliations:** 1 Dept. of Pathology and Molecular Medicine, Queen’s Cancer Research Institute, Queen’s University, Kingston, ON, Canada, K7L 3N6; 2 Dept. of Biomedical and Molecular Sciences, Queen’s Cancer Research Institute, Queen’s University, Kingston, ON, Canada, K7L 3N6; 3 Division of Cancer Biology and Genetics, Queen’s Cancer Research Institute, Queen’s University, Kingston, ON, Canada, K7L 3N6

## Background

The basal-like subtype of human breast cancers has no clinically defined marker profile. Recent studies have shown that Met as well as certain Src family kinases are over-expressed in basal-like breast cancers and correlate with disease progression and poor prognosis. Our group has also demonstrated a potential regulatory role of Stat3 on Met activation or turnover, adding to the previously identified roles of Stat3.

## Materials and methods

We have elected to test the efficacy of inhibitors of Met (PF-02341066), Src family kinases (Dasatinib), and Stat3 (CPA7) in blocking the invasive potential of breast cancer cells, using the human basal-like breast cancer cell line MDA-MB-231 transfected with a constitutively active Src mutant (MDA-Src cells). The efficacy of inhibitors in disrupting the invasive capacity of cells was assessed using transwell invasion assays and colony growth in a 3D Matrigel culture model mimicking basement membrane matrices.

## Results

Treatment of MDA-Src cells with Stat3 or Src inhibitors markedly reduced branching extensions, and induced growth arrest and cell death of colonies in a dose-dependent manner. Interestingly, treatment with the Met inhibitor also decreased branching extensions but failed to inhibit cell colony forming ability or proliferation. Furthermore, we have found that induction of morphological responses in 3-D culture occurs at ~15-fold lower concentration of the inhibitors than is necessary to observe decreased expression of drug targets in 2D culture. Transwell assays also showed a decrease in invasion of CPA7 and Dasatinib treated cells. Interestingly, PF2341066 treatment of cells did not affect transwell invasive potential, suggesting that other effectors than Met may be involved.

## Conclusions

Our findings further implicate a rate-limiting role of Src, Stat3 and Met in the process of invasion in human basal-like cells, and may lead to improved predictive biomarkers for targeting of aggressive breast cancer subtypes which exploit this pathway.

## Financial support

Terry Fox Transdisciplinary Studentship in Partnership with CIHR (EC), Ontario Graduate Studentship (EC), Canadian Breast Cancer Foundation (LR), and Canadian Institutes of Health Research (BEE).

